# Development of monoclonal antibodies against oropouche virus and its applicability to immunohistochemical diagnosis

**DOI:** 10.1186/s12985-024-02323-z

**Published:** 2024-04-08

**Authors:** Ana Paula Andreolla, Alessandra Abel Borges, Seigo Nagashima, Caroline Busatta Vaz de Paula, Lucia de Noronha, Nilson I.T. Zanchin, Juliano Bordignon, Claudia Nunes Duarte dos Santos

**Affiliations:** 1 Laboratório de Virologia Molecular, Instituto Carlos Chagas, ICC/Fiocruz PR, Cidade Industrial de Curitiba, Rua Prof. Algacyr Munhoz Mader 3775, Curitiba, Paraná Brazil; 2 Laboratório de Pesquisas em Virologia e Imunologia, Instituto de Ciências Biológicas e da Saúde (ICBS), Universidade Federal de Alagoas (UFAL), Av. Lourival Melo Mota, s/n, Tabuleiro do Martins, Maceió, Alagoas Brazil; 3 Laboratório de Patologia Experimental, Pontifica Universidade Católica do Paraná (PUC/PR), Rua Imaculada Conceição, 1155, Prado Velho, Curitiba, Paraná Brazil; 4 Laboratório de Biologia Estrutural e Engenharia de Proteínas, Instituto Carlos Chagas, ICC/Fiocruz PR, Cidade Industrial de Curitiba, Rua Prof. Algacyr Munhoz Mader 3775, Curitiba, Paraná Brazil

**Keywords:** Oropouche virus, Neglected virus, Monoclonal antibody, Immune-histochemistry diagnosis, Indirect immunofluorescent assay

## Abstract

*Orthobunyavirus oropouche ense virus* (OROV), the causative agent of Oropouche fever, is widely dispersed in Brazil and South America, causing sporadic outbreaks. Due to the similarity of initial clinical symptoms caused by OROV with other arboviruses found in overlapping geographical areas, differential diagnosis is challenging. As for most neglected tropical diseases, there is a shortage of reagents for diagnosing and studying OROV pathogenesis. We therefore developed and characterized mouse monoclonal antibodies and, one of them recognizes the OROV nucleocapsid in indirect immunofluorescent (IFA) and immunohistochemistry (IHC) assays. Considering that it is the first monoclonal antibody produced for detecting OROV infections, we believe that it will be useful not only for diagnostic purposes but also for performing serological surveys and epidemiological surveillance on the dispersion and prevalence of OROV in Brazil and South America.

## Introduction

*Orthobunyavirus oropouche ense virus* (OROV) is the etiological agent of a neglected emerging arboviral zoonotic disease named Oropouche fever, which is transmitted to humans mainly by *Culicoides paraensis* bites [1]. After OROV isolation in 1960 [2], several outbreaks of the Oropouche fever were reported in Brazil [3, 4]. After a long period without OROV disease notification, in 2006, the virus reemerged in Pará and Amazonas states, and came to be considered one of the most prevalent arboviruses in the Brazilian Amazon [5, 6] prior to the emergence of the zika and chikungunya viruses. Currently, four states of the North region in Brazil are experiencing outbreaks of OROV with a potential risk to other regions [7–9].

OROV is a member of the genus *Orthobynyavirus*, family *Peribunyaviridae* and comprises four genotypes, all of which are found in Brazil [4, 10]. The viral particle is spherical with a diameter of approximately 60 nm and a genome containing three negative segments of single-stranded genomic RNA (S, small; M, medium; L, large) surrounded by helical nucleocapsids. Each segment encodes different proteins, the nucleocapsid (N; 26.26 kDa) and the nonstructural protein s (NSs; 10.65 kDa) by the S segment, two structural surface glycoproteins (Gn with 32 kDa and Gc with 110 kDa) and one nonstructural protein m (NSm; 26.65 kDa) by the M segment, and the RNA-dependent RNA polymerase (261.25 kDa) by the L segment [11–15].

Clinical diagnoses of OROV infection are usually mistaken for diseases caused by other arboviruses that co-circulate in endemic areas, like dengue and yellow fever. Oropouche fever is described as an acute febrile illness commonly associated with headaches, fever, myalgia, and skin rash [2]. After infection, the OROV may also reach the central nervous system, resulting in rare cases of neurological syndromes and severe systemic infections but so far, there are no reports of sequelae resulting from the infection [16–19]. No licensed vaccines are currently available. The treatment for the Oropouche fever is based on the prescription of antipyretics and analgesics for symptom relief [20]. Laboratory diagnosis consists of molecular techniques (RT-PCR) and viral isolation in cultured cells, which are limited to the viremic phase [21–23].

Polyclonal and monoclonal antibodies (mAbs) are useful tools widely used in the serological diagnosis of infectious diseases [24–26]. In general, mAbs are more specific than polyclonal antibodies, reducing equivocation related to the interpretation of results and facilitating the standardization and reproducibility of the assays [27]. MAbs are also important for epidemiological surveillance, differential diagnosis, and pathogenesis studies [27]. Additionally, mAbs may have therapeutic properties and could eventually be used in immunotherapy, as has already been demonstrated for other viral infections [28]. In this study, we describe the development and characterization of mouse mAbs against a Brazilian strain of OROV and demonstrate its applicability as a diagnostic reagent in immunohistochemistry (IHC) assays.

## Animals and methods

### Cell line and viruses

C6/36 *Aedes albopictus* cells (ATCC CRL-1660) were grown in Leibovitz’s L15 medium (Gibco, Grand Island, USA) supplemented with 5% fetal bovine sera (FBS-Gibco), 25 µg/ml gentamicin (Gibco), and 0.27% tryptose at 28 °C. Vero E6 cells (Sigma, 85,020,206), A549 (ATCC CCL-185), Huh-7.5 (ATCC PTA-8561), and SH-SY5Y (ATCC CRL-2266) were cultured in Dulbecco’s Modified Eagle Medium/Nutrient Ham F12 (DMEM F12– Gibco) with 10% FBS, 14 mM sodium bicarbonate, 100 IU/ml penicillin (Sigma-Aldrich, Steinheim, Germany), and 100 µg/ml streptomycin (Sigma-Aldrich) at 37°c in a 5% CO_2_ atmosphere. Mouse myeloma cell line P3 × 63Ag8.653 (kindly supplied by Dr. Carlos R. Zanetti, from Laboratório de Imunologia Aplicada, Universidade Federal de Santa Catarina, Florianópolis, Brazil; ATCC CRL-1580) and hybridomas were maintained in RPMI-1640 medium (Cultilab, Campinas, Brazil) with 20% FBS, 23.8 mM sodium bicarbonate, 2 mM L-glutamine, 1 mM sodium pyruvate, 9.6 mM HEPES, 100 IU/ml penicillin, 100 µg/ml streptomycin, and 0.25 µg/ml amphotericin B (Sigma-Aldrich) at 37 °C in a 5% CO_2_ atmosphere.

OROV is a clinical isolate that was kindly supplied by Dr. Felipe Gomes Naveca [29] from Centro de Pesquisas Leonidas e Maria Deane, FIOCRUZ, Manaus, Amazonas, Brazil. It was allocated in Molecular Virology Laboratory (Instituto Carlos Chagas, FIOCRUZ, Curitiba, Paraná, Brazil), amplified in Vero E6, and titrated by the plaque-forming assay in C6/36 cells [50]. Dengue virus serotype 1 (strain FGA/89; DENV1), 2 (strain 265; DENV2) and 4 (strain 422; DENV4); zika virus (strain 15,261; ZIKV) and Mayaro virus (genotype D; MAYV) were amplified and titrated in C6/36 cell line.

### Immunization procedure

**Fig. 1:**
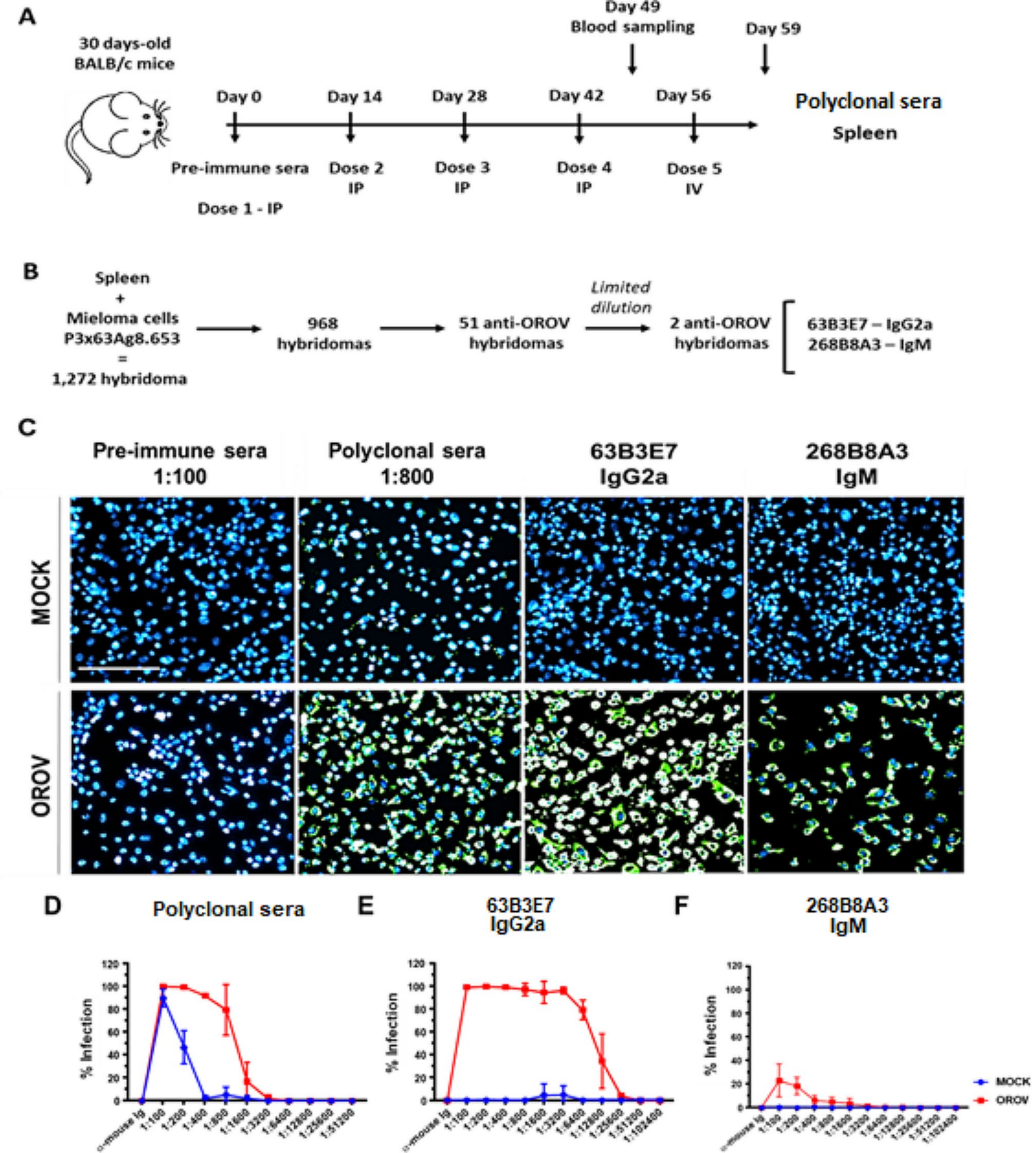
Production and selection of anti-OROV monoclonal antibodies. In **A**, scheme of inoculations in mice to produce mAbs, together with sera collection. In **B**, the screening of hybridomas resulting from the fusion is demonstrated, in addition to the following steps for selecting the most promising ones. In **C**, representative IFA for detection of anti-OROV antibodies from clones 63B3E7 (1:800) and 268B8E3 (1:100), together with the control sera (pre-immune and polyclonal, diluted at 1:100 and 1:800, respectively). The culture supernatant of the LD2 clones was purified and evaluated for the detection of viral antigens in C6/36 cells (1 × 10^4^ cells per well) uninfected (MOCK) and infected with OROV (MOI 1 for 48 h). In blue, cell nuclei stained with DAPI and in green OROV labeled with the respective mAb or polyclonal immune sera, followed by anti-mouse Ig conjugated with Alexa Fluor 488. In **D**–**F**, infected (OROV– red) and uninfected (MOCK - C6/36) cells– blue) were used for titration of polyclonal mouse sera (**D**), mAb 63B3E7 (**E**), and mAb 268B8A3 (**F**). The scale bar corresponds to 100 μm. **D**–**F** represents three experiments of three biological replicas

Three 30-day-old BALB/c mice were immunized five times each (14 days apart) with 1 × 10^6^ pfu C6/36/dose/animal with supernatant of OROV-infected cells mixed with Alu-S-Gel (Serva, Heidelberg, Germany) for doses 1 to 4 (intraperitoneal route); no adjuvant was included in the fifth dose (intravenous route) (Fig. 1A). Blood samples were collected prior to the first immunization (pre-immune sera) and after the fourth immunization by puncturing the caudal vein. The experimental protocol for animal use was approved by the Ethical Committee on Animal Research of the Fundação Oswaldo Cruz (Fiocruz CEUA no. LW-27/19).

### mAb production

Three days after administration of the fifth dose, the mice received an intraperitoneal anesthetic of 100 mg/kg ketamine/20 mg/kg xylazine, and the blood draw was performed by cardiac puncture to obtain post-immune sera. After bleeding, the mice were euthanized with an overdose of ketamine (500 mg/kg) and xylazine (100 mg/kg), and their spleens aseptically removed. The splenocytes were separated and fused with P3 × 63Ag8.653 cells using polyethylene glycol (MW 3000–3700; Sigma-Aldrich), as previously described [25]. The resulting hybrid cells (myeloma + splenocytes) were maintained in RPMI-1640 media, and hybridoma selection carried out using HAT media (RPMI supplemented with 100 µM hypoxanthine, 0.4 µM aminopterin and 16 µM thymidine; Sigma-Aldrich) for 14 days, followed by four days in HT media (RPMI supplemented with 100 µM hypoxanthine and 16 µM thymidine). Subsequently, hybridoma were maintained in RPMI media as previously described [25].

### Immunofluorescence assay

Hybridomas secreting anti-OROV antibodies were selected by indirect immunofluorescent assay (IFA) in OROV-infected C6/36 cells. Briefly, 1 × 10^4^ C6/36 cells/well (96-well plates) were infected with OROV at a multiplicity of infection (MOI) of 1 and fixed 48 h post-infection with methanol:acetone (v/v) overnight at -20 °C. After discarding the fixative reagent, the plates were dried in air, and 100 µl of hybridoma cell culture supernatant (from wells presenting cell grow) were added to each well and incubated for 1 h at 37 °C. After three washes with PBS-T (1x Phosphate Buffered Saline plus 0.05% Tween 20), anti-mouse IgG and IgM antibodies conjugated to Alexa Fluor 488 (A-10,680; Sigma-Aldrich; 1:1000 in 1% PBS-BSA) plus DAPI (0.3 mM) were added and incubated for 1 h at 37 °C. After three washes with 100 µl/well of PBS-T, the plates were analyzed under fluorescent microscopy. Images were captured by on an Operetta CLS High-Content Analysis System (PerkinElmer, Massachusetts, USA) using a 20x non-confocal objective and analyzed using the Harmony software (PerkinElmer). Nuclei quantification and percentage of cells infected by OROV were evaluated by staining with DAPI and Alexa Fluor 488, respectively. Data from infected cells were normalized according to their respective means of the positive infection control, which were considered 100% of marked cells, and of the MOCK (uninfected C6/36 cells), which we considered 0% of marked cells. The mice post-immune polyclonal OROV serum was used as a positive control for the staining, and the pre-immune serum was used as a negative control.

The supernatants of the clones that reacted to the infected cells and did not react to MOCK controls were subjected to limiting dilution (LD) to obtain a single cell per well. Upon reaching 75% confluence, IFA was performed again, and the clones whose supernatant still secrete antibodies which recognized OROV antigens in C6/36 infected cells were subjected to a second LD round. Once reactivity was confirmed again by IFA, isotyping was performed by immunoassay with the SBA Clonotyping System-HRP kit (SouthernBiotech, Birmingham,USA), as per the manufacturer’s recommendations. Subsequently, the hybridomas were expanded to produce mAbs, and cryopreserved (90% FBS plus 10% DMSO) for future use.

### Analysis of mAb reactivity to OROV antigens

The reactivity of the mAbs to OROV proteins was investigated using western blot assays. The supernatant of Vero E6 cells infected with OROV at MOI 0.001 was concentrated by polyethylene glycol (7% of PEG8000) precipitation and purified by sedimentation through a 30%/60% sucrose cushion in TNE (20 mM Tris pH 8.0, 150 mM NaCl, 2 mM EDTA). The cells were lysed (0.1% Triton-X, 150 mM NaCl, 50 mM Tris-HCl pH 8.0, 10% glycerol), centrifuged at 750 g for 1 min, and the supernatant was collected and stored at -80 °C. Purified viral supernatants, and infected and uninfected (MOCK) cell lysates were then diluted in Laemmli sample buffer (1:1), boiled for 5 min, and fractionated by SDS-PAGE (13% polyacrylamide) [26, 30].

Viral proteins were transferred to nitrocellulose membranes (6 mA for overnight in a cold-room) and blocked with 5% non-fat milk in TBS-T (20 mM Tris, 137 mM NaCl, pH 7.6 and 0.05% Tween 20) at room temperature for 1 h, followed by incubation with the mAbs 63B3E7 and 268B8A3, with the polyclonal antibody anti-OROV ascitic fluid (ATCC; VR 1228AF), and an isotype control (anti-envelope of flavivirus mAb 4G2). All antibodies were diluted 1:100 in 3 ml with 5% non-fat milk in TBS-T for 1 h at room temperature. After 3x washes with TBS-T 3 times for 5 min under agitation, the nitrocellulose membranes were incubated for 1 h at room temperature with an anti-mouse IgG secondary antibody alkaline phosphatase (AP) conjugate (cat number S3721, Promega, USA) or IgM (cat. number A9688, Sigma-Aldrich) diluted in 5% non-fat milk in TBS-T. The reaction was developed using the BCIP/NBT reagent (cat. number S3771, Promega, USA) according to the manufacturer’s recommendations.

### Identification of the OROV protein reacting with the 63B3E7 mAb by mass spectrometry

To identify the OROV protein reacting with the 63B3E7 mAb, two 13% polyacrylamide gels were performed in parallel using the same pre-stained protein mass standard (cat. number 26,616, Thermo Scientific). One gel was transferred to a nitrocellulose membrane and the band reacting with the 63B3E7 mAb was identified by western blot. The second gel was stained with Coomassie blue. Thus, based on the electrophoretic mobility of the protein recognized by the 63B3E7 mAb in the western blot (26 kDa), a band migrating in the same region of the Coomassie-stained gel was excised for mass spectrometry analysis. After separation of the proteins eluted from the affinity purification procedure by SDS-PAGE, each gel lane was split in three fractions, excised out of gel and each fraction was cut in 1 × 1 mm pieces. After destaining, proteins were reduced with 0.01 M DTT and alkylated with 0.05 M iodoacetamide before digestion using 12.5 ng/mL trypsin (Promega, V5113) diluted in 50 mM ammonium bicarbonate (ABC) at 37ºC for 18 h. Then, peptides were extracted from gel matrix, concentrated by vacuum centrifugation and desalted using C18 columns (Stagetip). Peptides were analyzed by LC-MS/MS using an Eksingent- nLC coupled online to an LTQ Orbitrap XL ETD (Thermo Scientific) (Mass Spectrometry Facility RPT02H / Carlos Chagas Institute - Fiocruz, Paraná, Brazil) [31]. Peptide samples were fractionated via reverse phase chromatography using a 15 cm fused silica capillary containing 3 μm C18 particles (ReproSil-Pur C18- AQ, Dr Maisch GmbH). The chromatography was carried out at 250 nL/min with a gradient of 5 to 40% of MeCN in 0.1% formic acid, 5% DMSO for 60 min [31]. Mass spectrometer was set to data-dependent acquisition mode, operating to alter automatically between MS1 and MS2 acquisition. MS1 spectra (m/z 300-2,000) were acquired in the Orbitrap analyser with a resolution of 60,000. The top 10 most intense precursor ions were sequentially isolated, fragmented by CID. OROV protein validation, quantification and identification used the MaxQuant platform (version 2.2.0.0) set to default parameters. Contaminant proteins (human keratins, BSA and porcine trypsin) and the reverse of all the sequences, including contaminants, were also included in the search and manually removed from the list of identifications.

### Immunohistochemistry assay

Three newborn BALB/C mice (first 48 h of life) were intracranially inoculated with 300 PFU of OROV or vehicle (MOCK mice) to evaluate the effectiveness of the mAbs in recognizing viral antigens in immunohistochemistry assays. After three days, the animals were euthanatized, and the brain tissues were collected and kept in buffered formalin solution (10% formaldehyde, 30 mM NaH_2_PO_4_ H_2_O, 45 mM NaH_2_PO_4_).

The anti-OROV clones 63B3E7 and 268B8A3, at 1:100 dilution, were used in the IHC assays of the mice-infected brain tissues. First, the anti-OROV mAbs were incubated overnight in a humid chamber in a temperature between 2 and 8ºC. After that, the secondary polymer (Mouse/Rabbit PolyDetector DAB HRP Brown, BSB0205, BioSB, Santa Barbara, CA) was applied to the tested material for 40 min at room temperature. The technique was revealed by adding the complex 2, 3, diaminobenzidine plus hydrogen peroxide substrate to develop the brown color, followed by counterstaining with Harris Hematoxylin. Two non-correlated mAbs that recognize flavivirus-specific E antigen (hybridoma D1-4G2-4-15, ATCC HB-112) and alphavirus-specific 1G1 (recognized CHIKV E1 envelope protein) were used as negative controls. The experimental protocol using animals was approved by the Ethical Committee on Animal Research of the Fundação Oswaldo Cruz (Fiocruz CEUA no. LW-20/20).

### Characterization of mAbs in different cell types infected by OROV

The reactivity of the anti-OROV 63B3E7 and 268B8A3 mAbs was evaluated by IFA in several cell lines infected by OROV (Vero E6, A549, Huh-7.5, and SH-SY5Y). Briefly, 2 × 10^4^ cells/well seeded in 96-well plates were infected with OROV (MOI of 0.02) and incubated at 48 h. At the end of the incubation period, the cells were fixed with 200 µl/well of methanol-acetone (1:1) at -20°C for 1 h. Next, 100 µl/well of anti-OROV mAbs 63B3E7 and 268B8A3, diluted at 1:800 and 1:100, respectively, in 1% PBS-BSA, was incubated for 1 h at 37°C. The mAbs dilutions were determined based on the results showed in the Fig. 1D. After three washes with PBS-T, a secondary anti-mouse IgG or IgM Alexa Fluor 488-conjugated antibody (1:1000; Sigma-Aldrich) and DAPI (0.3 mM; 4’,6-diamidino-2-phenylindole) diluted in 1% PBS-BSA were used to stain cells. The immunofluorescence images were acquired on an Operetta CLS High-Content Analysis System (PerkinElmer, Massachusetts, USA) using a 20x non-confocal objective and analyzed using the Harmony software (PerkinElmer).

### Evaluation of mAbs reactivity against different arboviruses

The reactivity of the anti-OROV 63B3E7 and 268B8A3 mAbs was evaluated by IFA against different arboviruses: dengue virus serotype 1 (strain FGA/89; DENV1), 2 (strain 265; DENV2) and 4 (strain 422; DENV4); zika virus (strain 15,261; ZIKV): Mayaro virus (genotype D; MAYV). Briefly, 2 × 10^4^ Huh-7.5 cells/well seeded in 96-well plates were infected at an MOI of 2 with DENV1, DENV2, DENV4, and ZIKV; an MOI of 0.5 of MAYV, and an MOI of 0.01 of OROV, and incubated at 48 h. At each time point, cells were fixed with 200 µl/well of methanol-acetone (1:1) at -20 °C for 1 h. The reactivity of the mAbs were determined by immunofluorescence assays as previously described in the section Immunofluorescence assay.

### Anti-OROV mAbs reactivity against hantavirus nucleocapsid protein

The reactivity of the anti-OROV mAbs against the hantavirus nucleocapsid protein was evaluated with a commercial enzyme immunoassay HANTEC (ICC/Fiocruz-PR) that detects IgG and IgM antibodies [32], as per manufacturer’s instructions. The mAbs 63B3E7 and 268B8A3 were diluted 1:100 in dilution buffer, and incubated 1 h at 37 °C. A labeled anti-mouse IgG (cat. number A4416) and IgM (cat. number A8786) peroxidase-conjugated were used as secondary antibodies, replacing the secondary human antibodies as the kit was developed for human use.

### Viral titration by plaque assay

The C6/36 cells were plated (1 × 10^5^ cells/well in 24 well plates) overnight, and infected (0.4 ml/well) with several serial dilutions (1:10) of OROV suspension (viral stock). After incubation for 1 h at 28 °C, the virus inoculum was discarded and cells washed once with 1x PBS. Then the cells were overlaid with a semi-solid culture medium (1:1 mixture of 3.2% CMC and L-15 media plus FBS and antibiotics) and incubated at 28 °C. After 7 days, the semi-solid medium was removed using 1x PBS (3x washes), cells were fixed with 3% paraformaldehyde and staining it with 2% violet crystal for 10 min. After washing thoroughly with water, the plaques formed were counted and the viral titer (PFU/ml) quantified using: 𝑻 = 𝑷 × 𝑰 × 𝑫. Where ***P*** is the number of plates counted; ***I*** is the volume of the inoculum in ml; and ***D*** corresponds to the dilution where plaque quantification was performed, and ***T*** the final titer of the virus.

## Results

### mAb production

Fusion of murine splenocytes with P3 × 63Ag8.653 cells resulted in 1,272 hybridomas. Of these, 968 wells were screened by IFA, and 148 (15%) were able to recognize OROV antigens in C6/36 infected cells, and not on MOCK cells. Positive clones were amplified and screened again by IFA resulting in 51 clones (34.5%) still secreting anti-OROV antibodies (Fig. 1B). The cells presenting the highest density and immunofluorescence intensity were subjected to LDs to obtain a single cell per well. After two successive LD cycles, two hybridomas secreting anti-OROV mAbs were obtained (Fig. 1B-C). The hybridomas were expanded in culture, and the supernatant from both clones was purified and concentrated. The mAb isotype was identified as IgG2a (clone 63B3E7) and IgM (clone 268B8A3), both with kappa light chains.

The specificity of the two anti-OROV antibodies was evaluated by IFA using C6/36 cells (Fig. 1D-F). Both mAbs 63B3E7 and 268B8A3 specifically reacted with OROV-infected cells (Fig. 1C-F), and no unspecific reaction was observed in MOCK-infected cells. Infected C6/36 cells showed similar staining pattern for the anti-OROV clone 63B3E7 and the post-immune mice polyclonal sera (1:800) (Fig. 1D-E).

### Analysis of mAb reactivity to OROV antigens

**Fig. 2:**
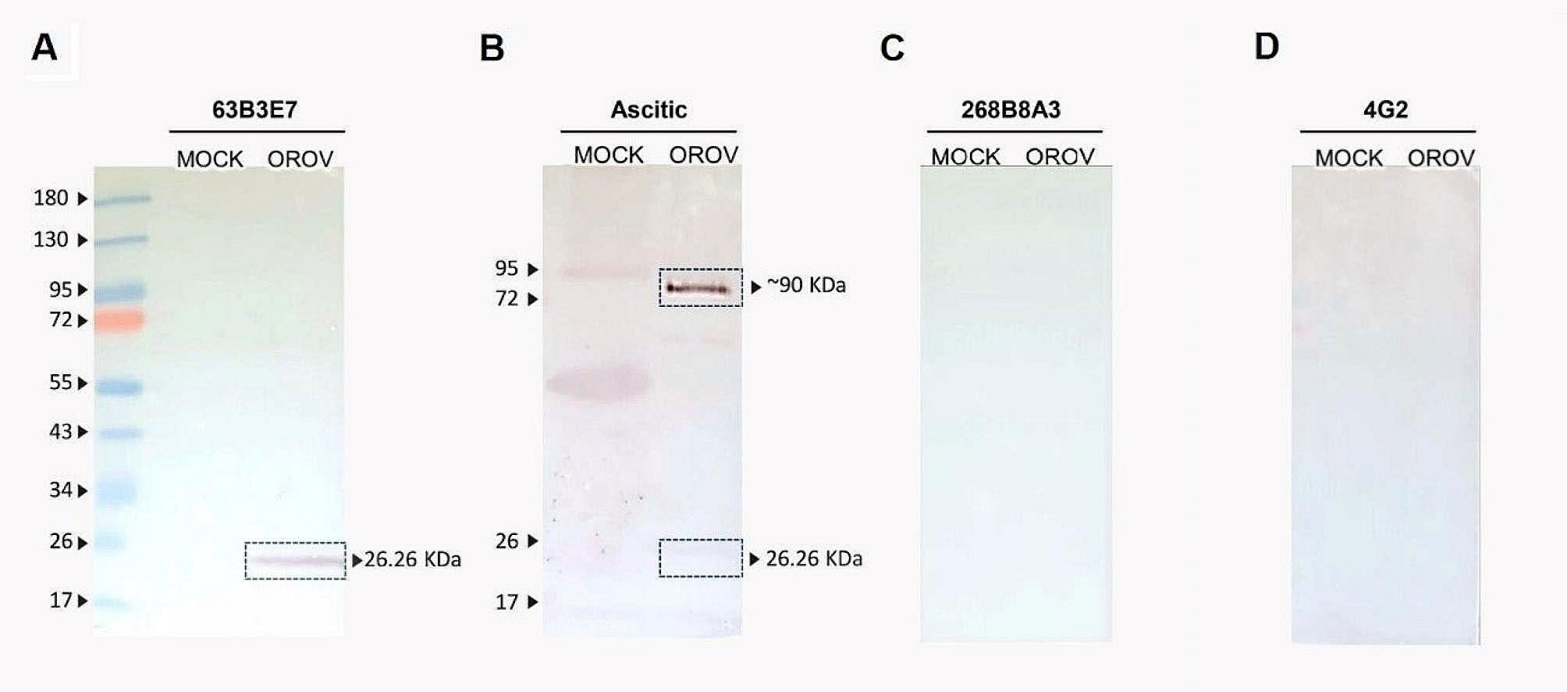
Western blots for the identification of the OROV protein target by the mAbs 63B3E7 and 268B8A3. OROV-infected Vero E6 cell purified (OROV) or MOCK reacted with anti-OROV mAbs 63B3E7 (**A**) and 268B8A3 (**C**). As controls an anti-OROV polyclonal ascitic fluid (**B**) and an anti-envelope of flavivirus mAb 4G2 (**D**). All were subjected to 13% SDS-PAGE and electroblotted onto nitrocellulose membranes. Proteins were stained with the mAbs, followed by an anti-mouse IgG or IgM conjugated to alkaline phosphatase. Molecular weight marker of 10–250 kDa

Infected and MOCK cell lysates and purified OROV supernatant were used in western blot assays to verify the identity of viral proteins recognized by both mAbs. The mAb 268B8A3 did not react with any protein in the samples tested in western blots, indicating that it might recognize only native proteins (Fig. 2C). On the other hand, the mAb 63B3E7 targeted a viral protein with electrophoretic mobility similar with the 26 kDa protein mass standard (Fig. 2A) in OROV-positive samples, consistent with the predicted molecular mass of the OROV nucleocapsid protein. The anti-OROV polyclonal ascitic fluid recognized the same 26 kDa and 90 kDa antigens on western blots (Fig. 2B). Also, the unrelated anti-flavivirus E-antigen specific mAb 4G2 did not recognized any OROV antigen in the same assay (Fig. 2D).

**Fig. 3:**
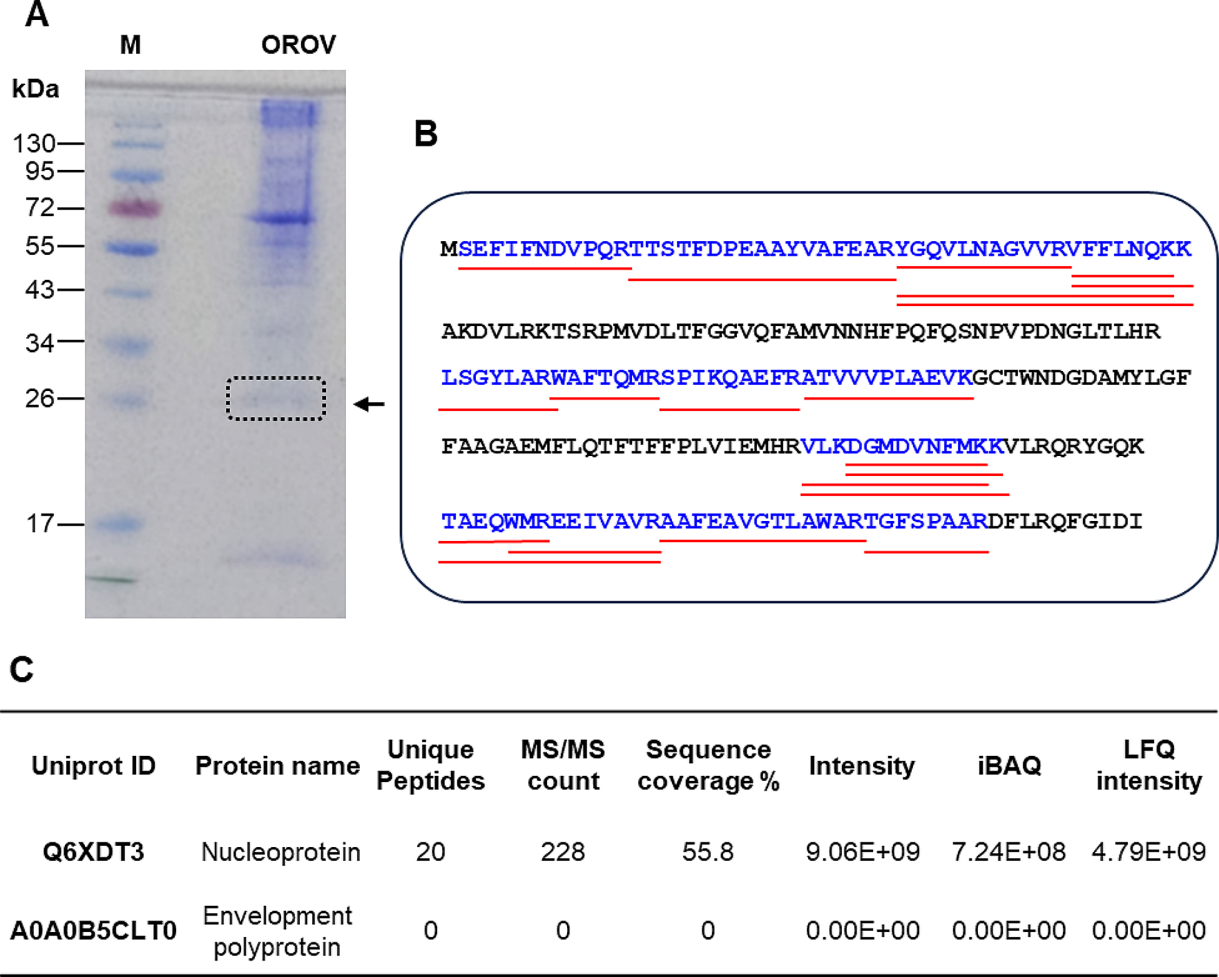
Mass spectrometry analysis of the OROV protein showing electrophoretic mobility of 26 kDa. **A**. Coomassie-stained polyacrylamide gel showing the protein mass standard (M) and the partially purified OROV proteins. The gel band excised for mass spectrometry analysis is indicated by a dotted box. **B**. Amino acid sequence of the OROV nucleocapsid protein (Uniprot ID Q6XDT3) showing in blue the region identified by mass spectrometry. The red lines underneath the amino acid sequence indicate the delimitations of the 20 unique peptides identified by mass spectrometry. **C**. Parameters obtained from the mass spectrometry analysis of the OROV protein showing electrophoretic mobility of 26 kDa

The identity of the OROV antigen detected by the mAb 63B3E7 was determined by mass spectrometry analysis of the OROV proteins migrating in the range of the 26 kDa protein mass standard. This analysis revealed 20 unique peptides of the OROV nucleocapsid protein, corresponding to 55.8% sequence coverage of the protein with Uniprot identification number Q6XDT3 (GenBank acession number AJT39491.1) (Fig. 3).

### Immunohistochemistry assay

**Fig. 4:**
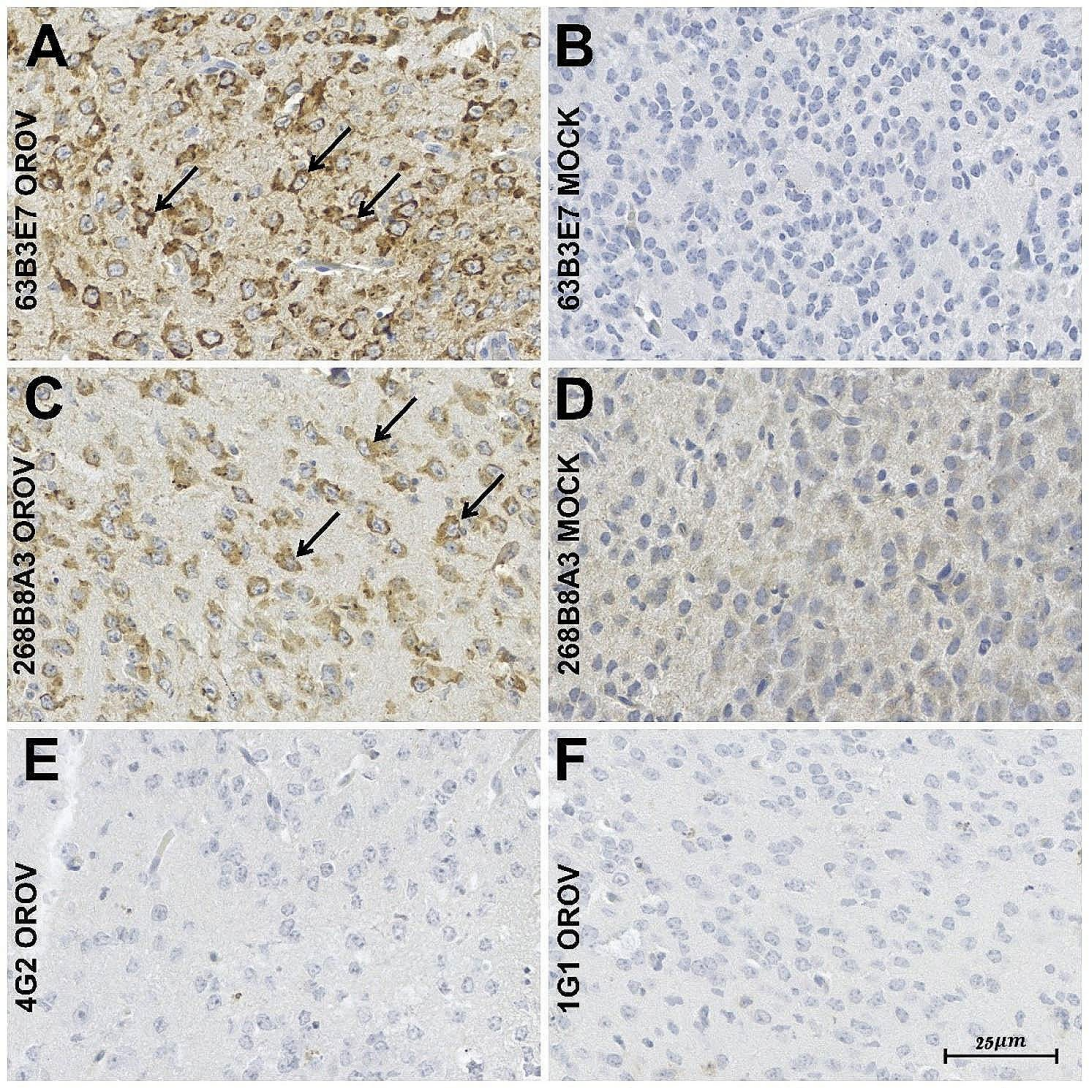
Immunohistochemistry in mouse neuronal tissue infected by OROV. Black arrows show perinuclear staining in neuronal tissue of mice infected by OROV and stained with mAb 63B3E7 (**A**) and mAb 268B8A3 (**C**). Image depicting the absence of perinuclear tissue and neuronal cytoplasmic immunolabel of the mAb 63B3E7 (**B**) and mAb 268B8A3 (**D**) in a neuronal tissue of mice uninfected with OROV (MOCK). E–F, negative control immunohistochemistry in OROV-infected tissue with mAbs 4G2 (anti-flavivirus), and 1G1 (Anti-alphavirus), respectively. The scale bar corresponds to 25 μm

The applicability of the two mAbs in IHC assays was evaluated. The 63B3E7 and 268B8A3 mAbs can differentiate between the OROV infection (Fig. 4A and C) and the MOCK cells (Fig. 4B and D) in mouse neuronal tissue. For both mAbs, the deposit of a granular brown stain (DAB), which is indicative of positive immunoreaction, was seen in the cytoplasm of cortical neurons, especially in the perinuclear region. The pyramidal cortical neurons and those located in the hippocampus presented the strongest positivity level of detection. No immunoreaction was observed in glial cells, endothelial cells, or any other cell types present in the mouse central nervous system. Although the mAbs were able to differentiate between infected and uninfected tissues, mAb 268B8A3 clearly produced a higher background in uninfected neuronal tissue at the dilution tested (Fig. 4D), compared to mAb 63B3E7. Mabs of the same isotype developed for other arboviruses (4G2 and 1G1) did not react to OROV proteins (Fig. 4E-F).

### Evaluation of mAbs reactivity in different cell types and against different viruses

**Fig. 5:**
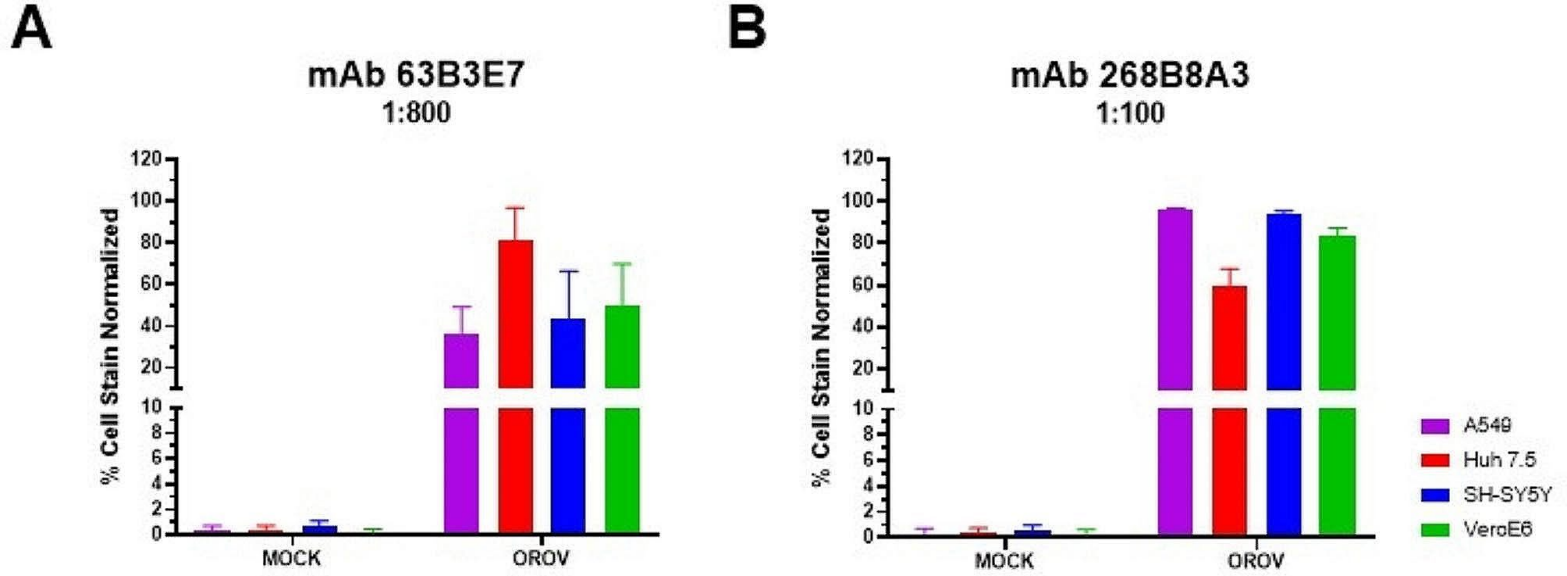
Reactivity of anti-OROV mAbs in different cell lines infected with OROV. The cell lines A549 (purple), Huh-7.5 (red), SH-SY5Y (blue) and Vero E6 (green) were infected with OROV (MOI of 0.01 for 48 h). After 48 h, the IFA assay was performed using anti-OROV 63B3E7 (**A**) and 268B8A3 (**B**) mAbs, followed by anti-mouse IgG and IgM conjugated to Alexa Fluor 488, respectively. The graphics show the normalized percentage of positive cells stained by each mAb in different cell lines infected by OROV. The graphics represent tree biological replica in triplicate

**Fig. 6:**
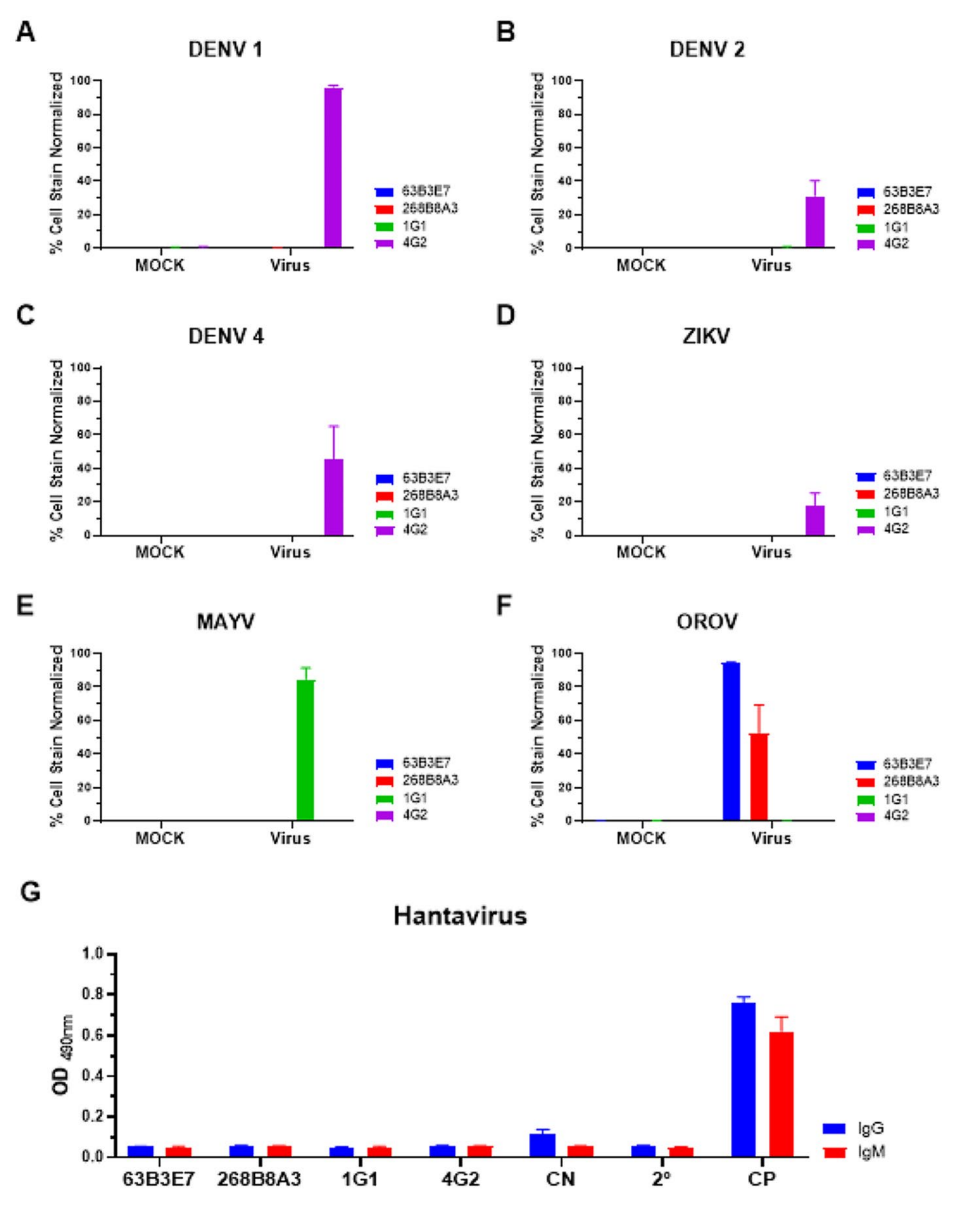
Reactivity of anti-OROV mAbs against different viruses. Huh-7.5 cell line (2 × 10^4^ cells per well) was infected with DENV1 (MOI of 2; **A**), DENV2 (MOI of 2; **B**), DENV4 (MOI of 2; **C**), ZIKV (MOI of 2; **D**), MAYV (MOI of 0.5; **E**), and OROV (MOI of 0.01; **F**). After 48 h, the IFA assay was performed using anti-OROV 63B3E7 (blue) and 268B8A3 (red) mAbs, anti-E protein of CHIKV, that cross recognize MAYV (1G1; green), and anti-envelope of flavivirus mAb 4G2 (4G2; purple), all diluted 1:100, followed by anti-mouse IgG and IgM conjugated to Alexa Fluor 488. In the graphics, normalized percentage cell stain data analyzed for each virus-infected Huh-7.5 cell line after 48 h. The EIA assay against the nucleocapsid protein of hantavirus (HANTEC) for detection of IgG (blue) and IgM (red) antibodies (**G**). In the graphic, the reactivity of anti-OROV mAbs (63B3E7 and 268B8A3) and controls anti-E protein of CHIKV (1G1) and anti-flavivirus family (4G2) were tested for both IgG (blue) and IgM (red). Negative and secondary antibodies (anti-IgG or IgM conjugated to HRP) were used as a control. As a positive control, an anti-nucleocapsid hantavirus mAb was used. The graphics represent one biological replica in triplicate

**Fig. 7:**
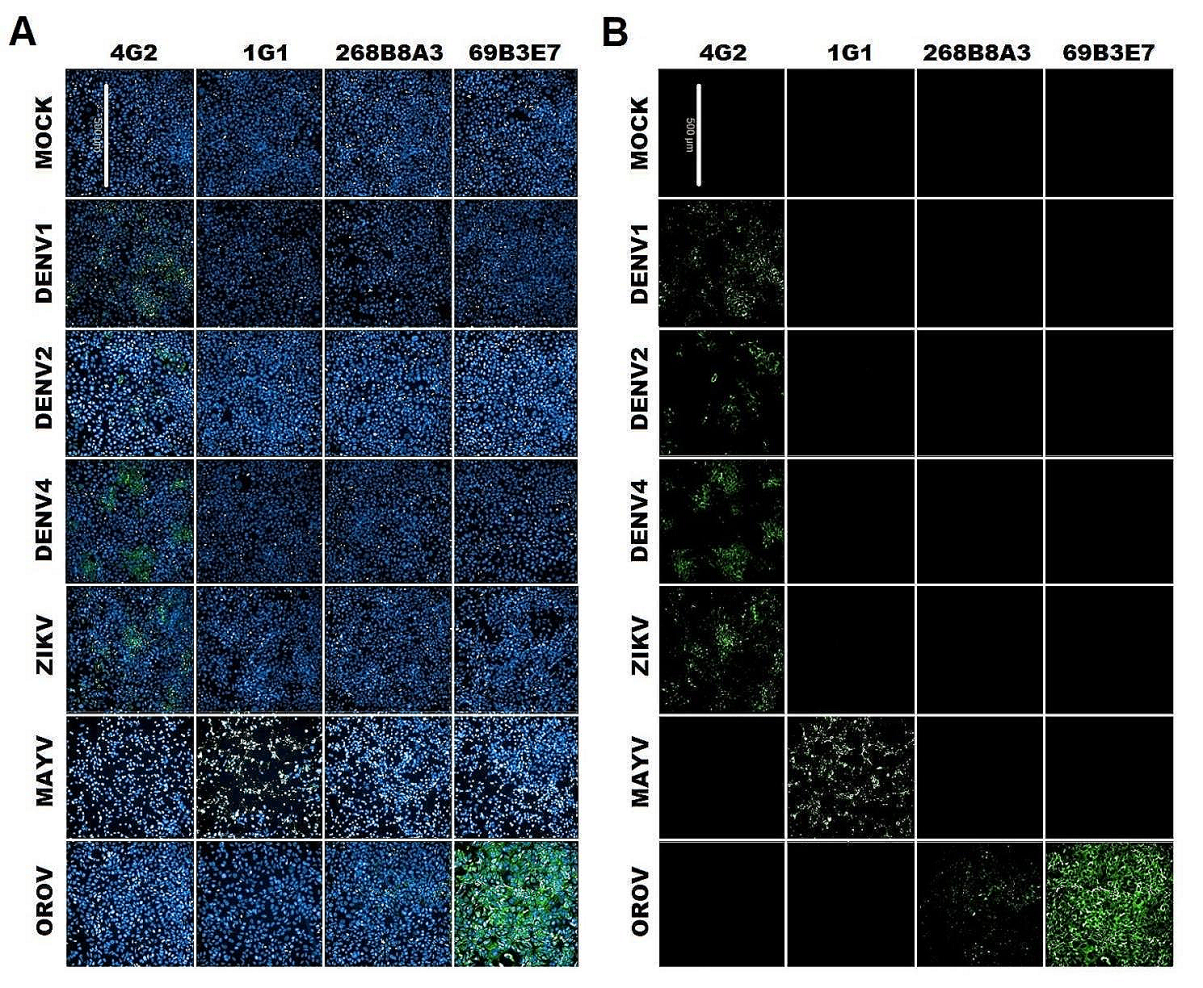
Reactivity of anti-OROV mAb against different arboviruses. Huh 7.5 cell line (2 × 10^4^ cells per well) was infected with DENV1 (MOI 2; **A**), DENV2 (MOI 2; **B**), DENV4 (MOI 2; **C**), ZIKV (MOI 2; **D**), MAYV (MOI 0.5; **E**) and OROV (MOI 0.01; **F**). After 48 h, the IFA assay was performed using anti-OROV 63B3E7 and 268B8A3 mAbs, anti-E protein of CHIKV that cross recognize MAYV (1G1) and anti-flavivirus family (4G2), followed by anti-mouse Ig conjugated to Alexa Fluor 488. In the images, MOCK represents uninfected cells after 48 h, and cells infected with respective virus for 48 h. As shows the merged image where in blue, nuclei stained with DAPI and, in green, OROV labeling through the mAbs was used, followed by anti-mouse IgG and IgM conjugated with Alexa Fluor 488 and in B, only stained by positive reaction with anti-mouse IgG and IgM conjugated with Alexa Fluor 488. The scale bar corresponds to 500 μm. Images are representative of one biological replica in triplicate

The reactivity of anti-OROV 63B3E7 and 268B8A3 mAbs was also analyzed in different cell lines, A549, Huh-7.5, Vero E6, and SH-SY5Y infected by OROV. The 63E3E7 and 268B8A3 mAbs specifically detected OROV-infected cells (Fig. 5). The cross-reactivity of these mAbs with other arboviruses of medical interest such as the dengue virus, zika virus, Mayaro virus, and hantavirus was also evaluated. Neither of the mAbs reacted with the tested viruses (Figs. 6 and 7).

## Discussion

OROV outbreaks occur in the Pará [5] and Amazonas [5, 33] states of Brazil and recently, cases of infection have been reported in other Brazilian regions, including the southeastern [19, 34], central [22] and northeast regions [21, 35]. Currently an OROV outbreak is affecting the Roraima, Amazonas, Rondonia and Acre States and more than 650 cases were confirmed in the first weeks of 2024 but, the Brazilian health services estimate that the OROV circulation might be broader [7–9].

Since the clinical symptoms of OROV infection are similar to those caused by other arboviral infections like dengue, especially in the early phase, the number of cases of OROV fever is underreported. As a neglected virus, few studies address the prevalence and dispersion of OROV and, its impact on the epidemiological panorama in Brazil and South America. The development of reagents, such specific mAbs, would therefore be relevant not only for diagnostic purposes but also for research use on viral biology, pathogenesis, and epidemiology, especially in countries where dengue and OROV are endemic and sympatric [22].

Currently, OROV diagnosis is essentially based on molecular tests, such as RT-PCR or real-time RT-PCR [19, 22, 36], as well as classical virology tests (viral isolation, hemagglutination inhibition, PRNT, and complement fixation tests) [3, 4, 23]. However, these are laborious, highly complex techniques that require specialized professionals to interpret the data. A simpler alternative would be the use of antibody-based tests. Currently, there are no diagnostic or reagents commercially available except, an ascitic fluid (ATCC; VR-1228AF) produced from animals infected with OROV [37], which has not been validated for diagnostic purposes. There are some studies employing OROV polyclonal antibodies [38, 39] but these reagents present variations from lot to lot that might impact the results. Therefore, developing specific and stable reagents, like anti-OROV mAbs, would guarantee the specificity and reproducibility of the assays [21, 27].

The two mAbs that we produced and characterized are able to differentiate OROV-infected cells or tissues from uninfected controls. Although both mAbs detected OROV infections, mAb 63B3E7 yielded better results both with IFA and IHC. In IFAs, mAb 63B3E7 specifically targets viral protein in all infected cell lines (lineages derived from humans, non-human primates, and mosquitoes) used in this study [40, 41].

The two mAbs are from different isotypes (63B3E7 is an IgG2a and 268B8A3 is an IgM) and they probably target different epitopes as both were reactive in IFA and IHC assays but only 63B3E7 was reactive in western blots [42]. The western blot and mass spectrometry analysis results suggest that mAb 63B3E7 recognizes a linear epitope of the nucleocapsid protein of OROV samples. These results are in line with those obtained for mAbs of the La Crosse and Tahyna viruses, which are viral species of the same family as OROV [45]. In a study using the La Crosse virus, the G2 protein ontologically corresponded to the Gn protein of OROV [42]. In denaturing western blot assays, the mAb 268B8A3 did not react with the proteins in the sample, possibly because it targets a conformational epitope [42].

Based on differences observed in the reactivity between the two anti-OROV mAbs produced here, they could be used for distinctive purposes. Considering that the mAb 63B3E7 recognizes a linear epitope of a conserved nucleocapsid protein, it would be useful for the development of immunoenzymatic assays. These assays are essential for viral diagnosis and epidemiological surveillance. It was already shown that monoclonal antibodies recognizing hantavirus nucleoprotein are useful tools for the development of a specific ELISA assay [25, 32]. On the other hand, as the anti-OROV mAb 268B8A3 seems to recognize a conformational epitope, it would represent a potential tool for functional studies both in vitro and in vivo, like neutralization assays. The ability of a monoclonal antibody to neutralize viral entry represent a relevant tool for pathogenesis studies and eventually for treatment of infected patients [28].

A percentage of OROV-infected patients can progress to neurological manifestations such as viral meningitis [17, 19], but the mechanism(s) involved is(are) unknown. Three studies [39, 46, 47] have developed murine models to study OROV infection in the nervous system and, all showed the OROV neural route and the ability to cross the blood-brain barrier [43, 44], triggering glial activation and neuronal cell death [35]. To investigate whether the mAbs that we developed could be useful in studying viral biology and for diagnosis using IHC, neuronal tissues of newborn mice experimentally infected with OROV were examined. Results showed positive immunostaining only in cortical neurons with both mAbs specifically in perinuclear intracytoplasmic, making the immunolocalization for the OROV-positive area reliable.

Despite the interesting reactivity pattern of both anti-OROV mAbs produced, it is important to mention that the mAbs were not tested against the four OROV genotypes. Thus, so far, we are not able to state that the anti-OROV mAbs detect all OROV genotypes circulating in South America. Nevertheless, the mAb 63B3E7 targets a linear epitope in the nucleocapsid protein, one of the most conserved protein for this virus family [44]. Therefore, despite the lack ok technical confirmation at this moment, it is plausible to consider that the mAb 63B3E7 would be able to recognize all OROV genotypes. An additional point is that due to lack of viral samples, it was not possible to test the mAbs’ reactivity against other related virus from the *Peribunyaviridae* family, which would be important to better understand the specificity of generated mAbs.

Summing up, we produced and characterized two mAbs, which specifically recognize OROV proteins and proved to be versatile and especially useful for differential diagnosis of OROV as well as to study basic aspects of OROV biology and pathogenesis. We anticipate that the mAbs developed in this work may contribute to support research, diagnosis, and epidemiological surveillance of OROV infections.

## Data Availability

Not applicable.
